# A State Hospital

**Published:** 1897-03

**Authors:** 


A State Hospital.—T. C. Thompson, Regent of the University of Texas (Medical Department) has published a "Brief Review of the Medical Department of the University of Texas" and the Journal is in receipt of a copy. After giving a detailed account of the conception, founding and development of the Medical Department Mr. Thompson says:

"The State owns the Sealy Hospital, which is a part of the Medical Department. It is at present leased at a nominal rental to the City of Galveston. It has accommodation for 125 patients and is constantly filled with patients from the city.

"There are always constant and increasing demands from the interior of the State for admission to the hospital from indigent sick, a number of whom evade the city laws and gain entrance, but the large majority of these cases can not be admitted. Further, the constant enlargement of the classes, and the increased demand for bedside instruction for the classes and for the physicians patronizing the School of Medicine, requires an increase in the number of patients and of the hospital facilities. It is recommended, therefore, that the State take up the lease from the city of Galveston and operate the hospital for the entire State, admitting all indigent sick from within her bounderies and collecting from counties sending such paupers to the hospital the cost of maintenance. A large part of the cost would be borne by the city of Galveston if arrangements be made with the city authorities to accommodate the city sick.

It should also be remembered that the asylum accommodations for the insane of Texas are inadequate for the number to be provided for, the county jails being occupied constantly by a greater or smaller number of such cases. An economy could well be practiced, and the congested condition of the asylums relieved, and at the same time the needs of the School served to advantage, by establishing several wards for insane cases under the same management as the general hospital, the officials of the School and the physicians of the hospital, as now constituted, being quiet sufficient, without the cost of the establishment and the maintenance of another asylum. Such a plan of a large State hospital has a distinguished precedent in the large Charity Hospital of Louisiana, which is maintained in New Orleans from special revenues of the State, as well as in many well known hospitals throughout the country, and benefices from State governments to subsidized hospitals, as in Pennsylvania and New York. The value of such increased hospital facilities to the School of Medicine would be beyond estimate, the most prominent objection to the institution being the meagerness of its clinical advantages. It is earnestly desired, therefore, that some relief or control of the hospital by the State as suggested above, be enacted at once."

The suggestion of Regent Thompson as regards making the Sealy hospital a State hospital and thus providing abundant clinical material for the school is an excellent one, and should be carried out. When we consider that the lack of clinical material and anatomical material has been a great disadvantage and hindrance to the growth of the Medical Department, its rapid growth becomes still more remarkable. On this point Mr. Thompson says:

"A better idea of this rapid growth may be had when it is stated that whereas in its first year the attendance was almost the lowest in the United States and Canada, it to-day ranks about twenty-fourth in attendance in the group of more than 120 medical colleges of this country and Canada.

"Nor are the schools, which have thus rapidly been passed in growth, inconspicuous and unworthy of regard; their number include such well known institutions as the medical departments of Yale University, Dartmouth College, Johns Hopkins University of Baltimore, University of Georgetown, University of Virginia, University of California, Western Reserve University of Cleveland, University of Colorado, Medical College of Virginia of Richmond, Medical College of South Carolina of Charleston, University of Buffalo, University of Missouri, and many others of as exellent reputation. All of the institutions which outrand this school in attendance are much older; many several generations older, some a century and a half older; many have much lower requirements and therefore attract a large but low grade class of students, but none can point to the same per centage increase in the last one or two years."

If such phenomenal development has been possible in the face of a palpable deficiency in clinical fatilities, it would be difficult to foretell the future growth of the Medical School, were all the pauper patients of the State sent to the hospital at Galveston.

The JOURNAL does not approve of Mr. Thompson's suggestion to have wards in the hospital for insane, with a view of relieving the crowded condition of the lunatic asylums. The State owes a duty to its insane population which should not be shirked nor halfway done. The accommodations for the insane in Texas are notoriously inadequate, there being more insane confined in jails and poor houses throughout the State than there are in the asylums. The best plan to relieve this is, beyond doubt, that suggested by Ex-Supt. F. S. White, of Terrell, Texas, that the State establish a farm or farms and build cottages on them for the use of the incurably insane, leaving the asylum for the recent cases or those supposed to be amenable to medical treatment. Dr. White's views in detail have more than once appeared in the TEXAS MEDICAL JOURNAL, and readers, if interested, are referred to the papers.

The suggestion, however, with regard to having a State hospital at Galveston, is, as we have said, sensible and timely, and the JOURNAL hopes to see it taken up and the necessary steps taken to carry it into effect.

